# IMPLEMENTATION OF A MINDFULNESS BASED DEMENTIA-CARE PROGRAM WITHIN A HEALTHCARE SYSTEM

**DOI:** 10.1093/geroni/igz038.1652

**Published:** 2019-11-08

**Authors:** Leah Hanson, Bhavani Kashyap, Michelle P Barclay

**Affiliations:** 1 HealthPartners Institute, St. Paul, Minnesota, United States; 2 The Barclay Group, LLC, Minneapolis, Minnesota, United States

## Abstract

The stress associated with caring for a loved one with Alzheimer’s disease or a related dementia can negatively affect mental and physical health. Mindfulness-based stress reduction (MBSR), teaching caregivers to focus on the present moment with non-judgmental awareness, has been shown to improve overall mental health, reduce perceived stress, and decrease depression. At HealthPartners, we implemented the Mindfulness Based Dementia-Care (MBDC) program, an 8-week program designed for family caregivers for those living with dementia. MBDC combines teaching of MBSR skills along with essential education for this chronic disease. Participants attend a 1-hour orientation session, 2-hour classes each week for 8 weeks, at-home practice between classes, and a 6-hour Saturday retreat. Secure electronic surveys are administered at baseline, program completion and follow-up for evaluation of the program. We will discuss barriers and learnings of implementation as well as preliminary results from the evaluation.

